# Peripheral Bone Removal versus Sequential Drilling Protocol in Dental Implant Surgery: A 5-Year Retrospective Study

**DOI:** 10.1055/s-0043-1772675

**Published:** 2023-11-23

**Authors:** Faaiz Yaqub Alhamdani, Ahmad Fliah Hassan, Hashim Mueen Hussein

**Affiliations:** 1 Department of Clinical Sciences, College of Dentistry, Ibn Sina University of Medical and Pharmaceutical Sciences, Baghdad, Iraq; 2 Department of Oral Medicine, College of Dentistry, Mustansiriyah University, Baghdad, Iraq; 3 Department of Conservative Dentistry, College of Dentistry, Mustansiriyah University, Baghdad, Iraq

**Keywords:** oral surgery, dental implant, sequential drilling, peripheral bone removal protocol

## Abstract

**Objective** The aim of this study was to compare the immediate success rate between peripheral bone removal (PBR) and conventional sequential drilling protocols.

**Materials and Methods** Biographic data of 130 Iraqi patients who attended a private dental implant center in Baghdad between January 7, 2018 and February 30, 2023 were collected. During this period, 198 dental implant procedures were completed. The recorded data included the zone of implantation, immediate or delayed implant, sinus lift procedure, dental implant system, bone augmentation, and dental implant length and diameter.

**Statistical Analysis** SPSS Ver. 25 was used for statistical analysis. Both descriptive and inferential statistics were applied.

**Results** In total, 198 dental implant procedures were performed during the study period. Of these, 104 cases were treated with the PBR protocol and 94 with the conventional drilling protocol. Out of 130 patients included in this study, 70 were treated with the PBR (IBS) technique and 60 patients were treated with the conventional dental implant systems. The early success of osseointegration reported in this study for all of the cases exceeded 93%. The PBR protocol was successful in 96 cases (92.3%), whereas early success of osseointegration in patients treated with the conventional protocol was reported in 89 cases (94.7%). The chi-squared test showed no statistically significant difference in the early success rate between the two dental implant protocols ( *p* = 0.575).

**Conclusion** In terms of immediate success, the PBR technique appears to be a reliable drilling technique. However, further longitudinal studies need to explore its potential to replace the sequential drilling protocol.

## Introduction

Over the past 50 years, dental implant practice is becoming more widespread worldwide. One of the main reasons for the popularity of dental implant is the high success rate of conventional dental implant systems. 1

The conventional dental implant approach follows the well-established protocol of sequential drilling, starting from the smallest diameter drill to the required drill diameter, 2 which matches the intended implant size. This protocol minimizes bone trauma through gradual bone removal to ensure optimum osseointegration.

Mechanical and thermal trauma during implant site preparation has been shown to play a crucial role in the failure of osseointegration. 3 This seems to encourage the tendency toward minimally invasive dentistry. 4 To achieve this aim, dental implant manufacturers started to propose new techniques, one of which uses a single-drill protocol. This protocol adopts specifically designed drills to eliminate the need for sequential drilling. It is time-saving and has less reported postoperative morbidity. 5

Peripheral bone removal (PBR) is one of the single-drill approaches that utilize the peripheral cut of the implant bony socket. 6 Despite this approach being in the market for more than a decade, little information is available on its early success rate compared with the conventional sequential drilling techniques. This study aims to compare the early success rate of PBR and conventional sequential drilling protocols.

## Materials and Methods

This study was approved by the Ethical Committee at Ibn Sina University of Medical and Pharmaceutical Sciences (ISU.2.1.23). This is a clinical retrospective study based on data from 130 Iraqi patients who attended a private dental implant center (Basmat Dental Center) in Baghdad between January 7, 2018 and February 30, 2023. During this period, 198 dental implant procedures were completed. These procedures were performed by the first author (F.A.) of the study. The recorded data were gender, age, zone of implantation (upper anterior, upper posterior, lower anterior, and lower posterior), immediate or delayed implant, dental implant side, sinus lift procedure, dental implant system, bone augmentation for the implant site, dental implant length, and dental implant diameter. Dental implant cases were either single implants or multiple implants (2–6 dental implants) for each patient.

**Fig. 1:**
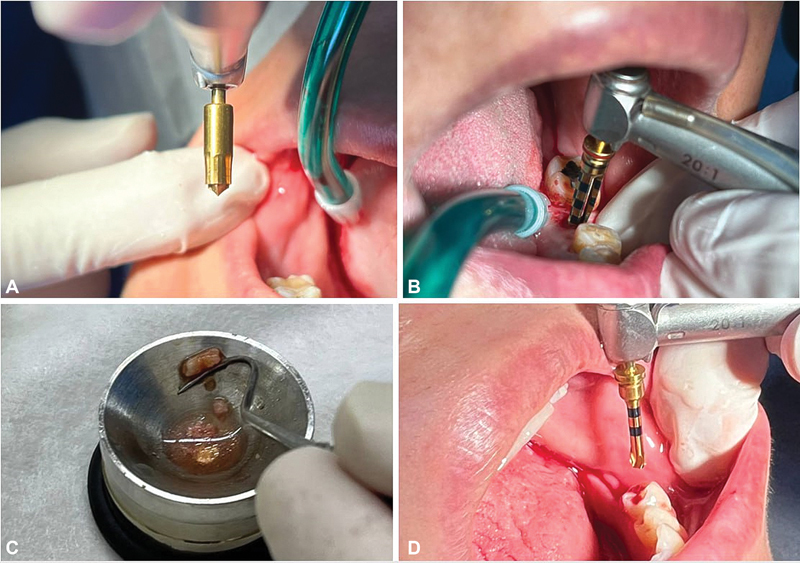
Peripheral bone removal (PBR) protocol. ( **A** ) Magic Marking drill for the preparation of the alveolar bone crest. ( **B** ) Implant bed preparation with the Magic Drill (IBS). ( **C** ) The bone core material retrieved from the implant socket bed after the PBR. ( **D** ) Magic Depth Drill (IBS) to level the socket floor.

All procedures with the PBR technique were performed in a flapless mode. The PBR protocol is a specific bone socket preparation protocol for dental implants. It uses a specifically designed hollow drill with three blades that remove the bone from the margin of the intended socket. These blades have sharp cutting ends and are joined near the cutting end. This drill does not remove the entire bone at the implant socket as in the conventional drilling protocols ( Fig. 1 ).

After the soft-tissue punch, the osteotomy entrance was prepared with the Magic Marking Drill (optional). The Magic Drill with a suitable diameter was used to prepare the implant bed ( Fig. 1 ). The drilling speed of 1,500 rpm can be used for the PBR technique. It is advisable to check the drill direction at the beginning of the drilling procedure (with a 3-mm depth). As it is a single-drill procedure, there is no chance of changing the direction as the surgeon continues the drilling. After reaching the required depth, a piece of the bone core is left. This piece either remains in the socket and is removed by means of a small excavator or can be retrieved using a small bone remover. Finally, the Magic Depth Drill was used to level the socket floor ( Fig. 1 ).

Antibiotics were not prescribed for the included patients. They were instructed to use a chlorhexidine digluconate mouthwash 0.12% for 2 minutes before the procedure and continue their mouthwash for 7 days postoperatively. The second-step surgery visit was performed 3 months after implant insertion visit. At this visit, the immediate success of osseointegration 7 8 was assessed on a clinical basis based on implant stability during cover screw removal and healing abutment placement. 9 10 11

The inclusion criteria were the following: dental implant cases performed using conventional (sequential) drilling and PBR 6 protocols. The exclusion criteria were cases with incomplete data, cases of immediate implants (where the PBR technique was not used), cases treated with the bone expansion by bending (BEB) technique, and cases in which a combination of the BEB and PBR techniques was used.

## Results

Table 1 provides the descriptive statistics of the study. The age range for the included patients was 21 to 78 years (mean: 46.97 years). In this study, the male-to-female ratio was approximately 1:2. Right side cases were more than left side cases. Similarly, the number of upper dental implants was higher compared with the number of lower dental implants, with the highest percentage reported for the upper posterior zone. The lowest percentage of dental implants was recorded in the lower anterior zone.

The length of the used dental implants ranged from 6 to 13 mm, whereas the dental implant diameters ranged from 2.5 to 7 mm. This range was the same for each of the dental arches. The shortest implant for the PBR technique was 7 mm, and the narrowest diameter of the implant for PBR was 3.5 mm.

Out of the total number of patients ( *n* = 130) included in this study, more than half of the patients ( *n* = 70) were treated with the IBS PBR technique, whereas the remaining ( *n* = 60) patients were treated with conventional dental implant systems. These systems were Dentaurum, ImplantKa, DeTech, Easy Implant, and NeoBiotech. The total number of dental implants used in this study was 198. One hundred and four implants were inserted using the PBR protocol, whereas 94 were inserted using the conventional drilling protocol.

More than one implant was needed in 34 patients. Twenty patients were treated by conventional dental implant systems, and 14 patients were treated with the IBS PBR technique. The number of multiple implants ranged between 2 and 6 dental implants for each patient.

Bone augmentation was used in 2 (1.9%) cases of the PBR technique and in 11 (11.7%) cases of the conventional technique. Sinus lift procedures were used in six (5.8%) cases of the PBR technique and in six conventional technique cases (6.4%).

**Fig. 2:**
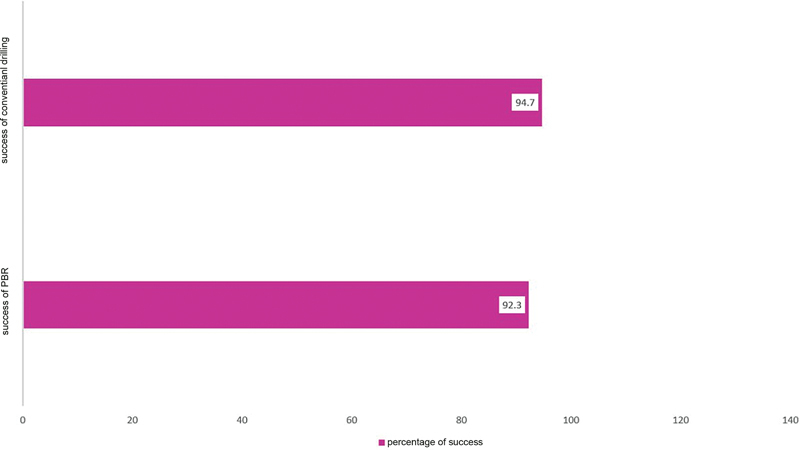
Level of early success in both peripheral bone removal (PBR) and conventional protocols.

In this study, the immediate-term success was reported in more than 93% of the cases. Dental implant with the PBR protocol was successful in 96 cases and success with the conventional protocol was reported in 89 cases ( Fig. 2 ). Chi-Squared Test showed no statistically significant difference between the success rates of the two dental implant surgical protocols.

## Discussion

The male-to-female ratio in this study indicates that dental implant treatment awareness was higher among females than among males. 12 13 14 15 16 This ratio may also partially explain the high percentage of dental implant treatment in the upper posterior zone. Females often request dental implant treatment for the upper first premolars for aesthetic reasons. 16 It is agreed that posterior teeth loss is higher than anterior teeth loss in both dental arches. 17 18 This might be the reason for the higher demand for posterior implants in both the upper and lower jaws. 10

The success rate reported in this study is comparable to other studies. 19 20 21 This study showed some differences in the number of bone augmentation cases between the two techniques. This could be attributed to the reliance of the surgeon on the bone graft material retrieved from the implant socket with the use of the PBR technique. This is one of the advantages of the PBR technique. The bone graft from the implant socket encompasses both osteoconductive and osteoinductive properties. 22

Autogenous bone graft is the gold standard in bone replacement, especially from intraoral sources. 23 The bone graft taken from the implant socket prepared by the PBR technique has an advantage over other intraoral sources, as additional site morbidity is avoided. Also, it is cost-effective, since bone augmentation materials from other sources are not needed.

Over the past 40 years, dental implant treatment using conventional sequential drilling protocols helped in achieving a high success rate. However, single-drill protocols can, also, help improve the treatment outcome. It overcomes the need for multiple drills, lengthy surgical procedures, related patient discomfort and prolonged tissue exposure with the related postoperative complaint, and increased chance of microbial contamination. 5 24 Moreover, single-drill protocols minimize the use of drills, which in turn will reduce the chance of drill wear, and subsequent bone trauma. 25

The PBR protocol is a brand-related technique (IBS). Each drill is designed for a specific fixture diameter (3.5, 4, 4.5, 5, 5.5, and 6 mm). Besides, it differs from the single-fluted drill protocols in terms of concept and drill design. Other single-drill techniques use specially designed tapered four-bladed drills. 5

Hence, it is difficult to compare PBR and single-fluted drills. Available studies on single-fluted drilling techniques both *in vitro* and clinical studies evaluated different aspects of single-drill techniques using fluted techniques (OsseoFuse/OneDrill, IdAll Implants, and Diffusion International). 24 26 27 Available literature has not provided a comparison between the PBR technique and other drilling techniques, whether conventional or single fluted drills, in terms of the accuracy of the implant procedures.

Gehrke et al conducted comparative studies both *in vitro* and clinical. They found that a singular drill system provides more precise osteotomy than conventional sequential drilling systems. 26 In another preclinical study, they did not find a difference regarding the peri-implant bone behavior between sequential and single drill techniques. 27

In their randomized controlled trial (RCT) study, Guazzi et al assessed the 4-month postoperative success of both sequential drill and single-drill protocols in 40 patients (20 patients in each protocol). They compared the outcome of the procedures using the following criteria: implant failure; complications; marginal bone loss; operation duration; and operator preference and postoperative pain, swelling, and the need for analgesic. They found a similar level of success with the advantage of shorter procedures with fewer postoperative complaints in the single-drill technique. 5

The level of immediate-term success reported in the PBR technique could be related to the hollow drill design, which seems to minimize both mechanical and thermal bone trauma during the implant socket preparation. Single use of drills also decreases the chance of additional bone trauma, an advantage reported with other single-drill techniques. 24 26 27

The PBR technique did not get enough attention from researchers. Being a single-drill-based technique, it is a time-saving socket bed preparation technique with minimal patient discomfort. To the best of the authors' knowledge, this is the first clinical retrospective study that compares the PBR protocol with the conventional drilling protocol. So far, only one case series on the PBR technique has been published, which was done by Senada et al. 28 However, this study did not make any comparison between different techniques and the sample size was limited.

The main limitation of this study is its retrospective nature, which limits the control of the researchers over the variables. Prospective longitudinal studies are required for long-term assessment of the PBR drilling protocol.

To conclude, in terms of immediate-term clinical success, the PBR technique appears to be a reliable drilling technique. However, further longitudinal studies need to explore its potential to replace the sequential drilling protocol.

**Table 1:** Study's descriptive statistics

Variable	*N*	Percentage
Males	69	34.8
Females	129	65.2
**Dental implant side**		
Right side	111	56.1
Left side	87	43.9
**Upper vs. lower jaw**		
Upper	112	56.6
Lower	86	43.4
**Implant zone**		
Upper anterior	33	16.7
Upper posterior	82	41.4
Lower anterior	7	3.5
Lower posterior	76	38.4
**Early success rate**		
Successful	185	93.4
Failure	13	6.6
**Drilling protocol**		
PBR technique	104	52.5
Conventional technique	94	47.5

Abbreviation: PBR, peripheral bone removal.

## References

[1] Moraschini V Poubel L A Ferreira V F Barboza EdosS Evaluation of survival and success rates of dental implants reported in longitudinal studies with a follow-up period of at least 10 years: a systematic review Int J Oral Maxillofac Implants 2015 44 03 377 388 10.1016/j.ijom.2014.10.023 25467739

[2] Albrektsson T Brånemark P I Hansson H A Lindström J Osseointegrated titanium implants. Requirements for ensuring a long-lasting, direct bone-to-implant anchorage in man Acta Orthop Scand 1981 52 02 155 170 7246093 10.3109/17453678108991776

[3] Marheineke N Scherer U Rücker M Evaluation of accuracy in implant site preparation performed in single- or multi-step drilling procedures Clin Oral Investig 2018 22 05 2057 2067 10.1007/s00784-017-2312-y 29250716

[4] Ericson D What is minimally invasive dentistry? Oral Health Prev Dent 2004 2(1, Suppl 1):287 292 15646587

[5] Guazzi P Grandi T Grandi G Implant site preparation using a single bur versus multiple drilling steps: 4-month post-loading results of a multicenter randomised controlled trial Eur J Oral Implantology 2015 8 03 283 290 26355172

[6] IBS Magic FC Implants 2017 https://ibsimplant.us/innovative-solutions/magic-fc-implants/

[7] Nilawati N Widyastuti W Rizka Y Kurniawan H Dental implant osseointegration inhibition by nicotine through increasing nAChR, NFATc1 expression, osteoclast numbers, and decreasing osteoblast numbers Eur J Dent 2023 17 04 1189 1193 36574781 10.1055/s-0042-1758794 PMC10756838

[8] Ustun Y Erdogan O Kurkcu M Akova T Damlar I Effects of low-intensity pulsed ultrasound on dental implant osseointegration: a preliminary report Eur J Dent 2008 2 04 254 262 19212531 PMC2634779

[9] Elias C N Meirelles L Improving osseointegration of dental implants Expert Rev Med Devices 2010 7 02 241 256 20214429 10.1586/erd.09.74

[10] Alhamdani F Abdullah E The influence of local factors on early dental implant failure, 5-year retrospective study J Odontol Res 2021 9 01 5 10

[11] French D Larjava H Ofec R Retrospective cohort study of 4591 Straumann implants in private practice setting, with up to 10-year follow-up. Part 1: multivariate survival analysis Clin Oral Implants Res 2015 26 11 1345 1354 25134415 10.1111/clr.12463

[12] Grisar K Sinha D Schoenaers J Dormaar T Politis C Retrospective analysis of dental implants placed between 2012 and 2014: indications, risk factors, and early survival Int J Oral Maxillofac Implants 2017 32 03 649 654 28212455 10.11607/jomi.5332

[13] Jang H-W Kang J K Lee K Lee Y S Park P K A retrospective study on related factors affecting the survival rate of dental implants J Adv Prosthodont 2011 3 04 204 215 22259704 10.4047/jap.2011.3.4.204 PMC3259446

[14] Geckili O Bilhan H Geckili E Cilingir A Mumcu E Bural C Evaluation of possible prognostic factors for the success, survival, and failure of dental implants Implant Dent 2014 23 01 44 50 24113554 10.1097/ID.0b013e3182a5d430

[15] Kang D-Y Kim M Lee S J Early implant failure: a retrospective analysis of contributing factors J Periodontal Implant Sci 2019 49 05 287 298 31681486 10.5051/jpis.2019.49.5.287 PMC6819696

[16] Alhamdani F Abdulla E HJJMR Sciences H Influence of patient's age and gender on dental implant treatment five year retrospective study J Med Res Health Sci 2021 4 09 1461 1467

[17] Zhang Z Y Meng T Chen Q Liu W S Chen Y H Retrospective analysis of early dental implant failure Beijing Da Xue Xue Bao 2018 50 06 1088 1091 30562787

[18] Schoenbaum T R Moy P K Aghaloo T Elashoff D Risk factors for dental implant failure in private practice: a multicenter survival analysis Int J Oral Maxillofac Implants 2021 36 02 388 394 33909732 10.11607/jomi.8983

[19] Chrcanovic B R Kisch J Albrektsson T Wennerberg A Factors influencing early dental implant failures J Dent Res 2016 95 09 995 1002 27146701 10.1177/0022034516646098

[20] Baqain Z H Moqbel W Y Sawair F A Early dental implant failure: risk factors Br J Oral Maxillofac Surg 2012 50 03 239 243 21612850 10.1016/j.bjoms.2011.04.074

[21] Olmedo-Gaya M V Manzano-Moreno F J Cañaveral-Cavero E de Dios Luna-del Castillo J Vallecillo-Capilla M Risk factors associated with early implant failure: a 5-year retrospective clinical study J Prosthet Dent 2016 115 02 150 155 26545864 10.1016/j.prosdent.2015.07.020

[22] Karalashvili L Kakabadze A Uhryn M Vyshnevska H Ediberidze K Kakabadze Z Bone grafts for reconstruction of bone defects Georgian Med News 2018 282 44 49 30358539

[23] McKenna G J Gjengedal H Harkin J Holland N Moore C Srinivasan M Effect of autogenous bone graft site on dental implant survival and donor site complications: a systematic review and meta-analysis J Evid Based Dent Pract 2022 22 03 101731 36162883 10.1016/j.jebdp.2022.101731

[24] Chen L A one-drill system for predictable osteotomy and immediate implant placement EC Dent Sci 2023 22 114 128

[25] Koopaie M Kolahdouz S Re: Heat generation and drill wear during dental implant site preparation Br J Oral Maxillofac Surg 2017 55 09 985 10.1016/j.bjoms.2017.08.372 29017733

[26] Gehrke S A Guirado J LC Bettach R Fabbro M D Martínez C P Shibli J A Evaluation of the insertion torque, implant stability quotient and drilled hole quality for different drill design: an *in vitro* Investigation Clin Oral Implants Res 2018 29 06 656 662 26957224 10.1111/clr.12808

[27] Gehrke S A Bettach R Aramburú Júnior J S Prados-Frutos J C Del Fabbro M Shibli J A Peri-implant bone behavior after single drill versus multiple sequence for osteotomy drill BioMed Res Int 2018 2018 9.756043E6 10.1155/2018/9756043 PMC5925187 29850594

[28] Senada E A El Sheikh S A Khalil M MJADJ Evaluation of success of single drilling implant system (clinical and radiographic study) Alex Dent J 2020 45 02 60 66

